# Supplementary material for: EnzML: multi-label prediction of enzyme classes using InterPro signatures
Source: BMC Bioinformatics. 2012 Apr 25;13:61. doi: 10.1186/1471-2105-13-61 (PMC3483700; doi:10.1186/1471-2105-13-61)
Supplement: Addtional file 5 — The Java code to format the data files, evaluate and predict. The file enzml_java_code.tar.gz contains the Java code used to format database data to ARFF and XML formats, to execute cross and train-test (jackknife) evaluations and to record evaluation results to database. More information is included in the readme.txt file and the Javadoc files. The code can be used with a MySQL database. To use a different database software, other JDBC drivers might be required. [file 1471-2105-13-61-S5.gz › java_code/enzml2011/doc/uk/ac/ed/inf/enzml/mulan/MulanArff.html]

MulanArff


---


|  |  |  |
| --- | --- | --- |
| **Overview** \| **Package** \| **Class** \| **Use** \| **Tree** \| **Deprecated** \| **Index** \| **Help** | |  |
| **PREV CLASS**   **NEXT CLASS** | **FRAMES**    **NO FRAMES**     **All Classes** |  |
| SUMMARY: NESTED \| FIELD \| CONSTR \| METHOD | DETAIL: FIELD \| CONSTR \| METHOD |  |


---


## uk.ac.ed.inf.enzml.mulan Class MulanArff

```
java.lang.Object
  uk.ac.ed.inf.utils.Initialised
      uk.ac.ed.inf.enzml.weka.Arff
          uk.ac.ed.inf.enzml.mulan.MulanArff
```

**All Implemented Interfaces:**: uk.ac.ed.inf.utils.Initialisable

**Direct Known Subclasses:**: AttributesFilteredArff

---

``` public class MulanArff extends Arff ```

Generates an Attribute Relation Format File (ARFF) in Mulan format (multiple
class labels per instance). The class attributes must be in a separate Mulan
XML file (either flat or hierarchical) to distinguish them from the normal
attributes.
Call structure: the MulanArffGenerator creates a DataSetManager which checks
the input (DataSetChecker), loads the data (MulanDataSetDbLoader), creates
the instances (MulanDataSetGenerator) and writes them as ARFF file
(DataSetWriter).

**Version:**
:   10 May 2010

**Author:**
:   Luna De Ferrari luna.deferrari-at-ed.ac.uk

---

| **Field Summary** | |
| --- | --- |
| `boolean` | `m_memoryAbundant`             if plenty of memory available, also load each instance in weka instances, otherwise only write them to file one by one |

**Fields inherited from class uk.ac.ed.inf.utils.Initialised**

`m_initialisationIsCorrect`


**Constructor Summary**

`MulanArff(java.lang.String dataPropsFileName,
java.lang.String resultsPropsFileName)`

`MulanArff(java.lang.String dataPropsFileName,
java.lang.String resultsPropsFileName,
boolean memoryAbundant)`


| **Method Summary** | |
| --- | --- |
| `MulanDataSetManager` | `getDataSetManager()` |
| `weka.core.Instances` | `getInstances()` |
| `java.util.Vector<java.lang.String>` | `getInstancesNames()` |
| `java.lang.String` | `getXmlPath()` |
| `static java.lang.String` | `getXMLpathFromArffPath(java.lang.String arffPath)`             Generates the xml file path: same name+path as .arff file, but .xml termination |
| `static void` | `main(java.lang.String[] args)` |
| `static int[]` | `run(java.lang.String[] arffPaths, java.lang.String machineLearningProps)`             Generates a number of ARFF files (the database the records are written is the same for all ARFF files) |
| `static int` | `run(java.lang.String arffPropsPath, java.lang.String machineLearningProps)`             Generates the ARFF (and XML) files and returns the identifier of the arff record in the database |
| `void` | `setDataSetManager()` |

**Methods inherited from class uk.ac.ed.inf.enzml.weka.Arff**

`checkAndInitialise, fillArffButDontWrite, generateAndWriteArffFile, generateArff, getArffPath, getArffRecordId, getDataArffProperties, getDataDbManager, getDataPropsFilePath, getDbConn, getDbReader, getResultsDbManager, memoryIsAbundant, setArffRecordId`

**Methods inherited from class uk.ac.ed.inf.utils.Initialised**

`initialisationIsCorrect, setInitialisationIsCorrect`

**Methods inherited from class java.lang.Object**

`equals, getClass, hashCode, notify, notifyAll, toString, wait, wait, wait`

**Field Detail**

### m\_memoryAbundant

```
public boolean m_memoryAbundant
```

:   if plenty of memory available, also load each instance in weka instances,
    otherwise only write them to file one by one


**Constructor Detail**

### MulanArff

```
public MulanArff(java.lang.String dataPropsFileName,
                 java.lang.String resultsPropsFileName)
```

---


### MulanArff

```
public MulanArff(java.lang.String dataPropsFileName,
                 java.lang.String resultsPropsFileName,
                 boolean memoryAbundant)
```


**Method Detail**

### getDataSetManager

```
public MulanDataSetManager getDataSetManager()
```

:   **Overrides:**: `getDataSetManager` in class `Arff`

---


### getInstancesNames

```
public java.util.Vector<java.lang.String> getInstancesNames()
```

---


### getXmlPath

```
public java.lang.String getXmlPath()
```

---


### getInstances

```
public weka.core.Instances getInstances()
```

:   **Overrides:**: `getInstances` in class `Arff`

---


### setDataSetManager

```
public void setDataSetManager()
```

:   **Overrides:**: `setDataSetManager` in class `Arff`

---


### getXMLpathFromArffPath

```
public static java.lang.String getXMLpathFromArffPath(java.lang.String arffPath)
```

:   Generates the xml file path: same name+path as .arff file, but .xml
    termination

    :   **Parameters:**: `arffPath` - the path to the arff file **Returns:**: the same filename+directorypath of the arff file but with the termination .xml instead of .arff

---


### main

```
public static void main(java.lang.String[] args)
```

---


### run

```
public static int run(java.lang.String arffPropsPath,
                      java.lang.String machineLearningProps)
```

:   Generates the ARFF (and XML) files and returns the identifier of the arff
    record in the database

    :   **Parameters:**: `arffPropsPath` - the path of the ARFF properties file (queries that define the data to be included): `machineLearningProps` - the database connection to the database containing the arff file table, to save the arff file details. **Returns:**: the identifier of the generated ARFF file in the database table

---


### run

```
public static int[] run(java.lang.String[] arffPaths,
                        java.lang.String machineLearningProps)
```

:   Generates a number of ARFF files (the database the records are written is
    the same for all ARFF files)

    :   **Parameters:**: `arffPaths` - a list of arff properties files (containing the data queries): `machineLearningProps` - the database connection properties file to write the arff records too **Returns:**: a list of arff records identifiers in the database arff table


---


|  |  |  |
| --- | --- | --- |
| **Overview** \| **Package** \| **Class** \| **Use** \| **Tree** \| **Deprecated** \| **Index** \| **Help** | |  |
| **PREV CLASS**   **NEXT CLASS** | **FRAMES**    **NO FRAMES**     **All Classes** |  |
| SUMMARY: NESTED \| FIELD \| CONSTR \| METHOD | DETAIL: FIELD \| CONSTR \| METHOD |  |


---
